# Tricuspid Papillary Fibroelastoma: A Rare Tumor in an Uncommon Location

**DOI:** 10.7759/cureus.61709

**Published:** 2024-06-05

**Authors:** Hanad Bashir, Ahmed Ahmed, Gauranga Mahalwar, Jason Lane, Adeeb Alquthami

**Affiliations:** 1 Cardiovascular Medicine, The Christ Hospital, Cincinnati, USA; 2 Internal Medicine, Unity Hospital, Rochester, USA; 3 Internal Medicine, Cleveland Clinic Akron General, Akron, USA; 4 Pathology, Cleveland Clinic Akron General, Akron, USA; 5 Cardiology, Cleveland Clinic Akron General, Akron, USA

**Keywords:** cardiac papillary fibroelastoma, imaging, tricuspid valve, echocardiography, cardiac magnetic resonance

## Abstract

Papillary fibroelastomas (PFEs) are rare benign cardiac tumors typically arising from the valvular endocardium, often affecting the aortic and mitral valves. They can range from asymptomatic to causing severe thromboembolic complications like stroke. This article presents a case of a tricuspid valve PFE in an 81-year-old patient with severe multi-vessel coronary artery disease. Transthoracic echocardiography revealed a mass on the tricuspid valve, confirmed by cardiac MRI. The patient underwent surgical excision, and histopathology confirmed the PFE diagnosis. The case highlights the importance of multimodal imaging in diagnosis and individualized treatment strategies for valvular heart tumors.

## Introduction

Papillary fibroelastomas (PFEs) represent an exceptionally rare subset of benign cardiac tumors, primarily originating from the valvular endocardium. Despite their infrequency, PFEs present a broad spectrum of clinical manifestations, ranging from asymptomatic incidental findings to severe thromboembolic complications. This diagnostic challenge arises from the diverse clinical presentations and imaging characteristics associated with these tumors [1]. An understanding of the epidemiology and clinical manifestations of PFEs is imperative for prompt diagnosis and effective management. Therefore, this case report aims to elucidate a pathologically confirmed instance of tricuspid PFE, delineating its detection through a comprehensive diagnostic approach integrating transthoracic echocardiography (TTE) and cardiac magnetic resonance imaging (CMRI). By exploring the nuances of PFEs, including their incidence, presentation, and diagnostic modalities, we seek to provide clinicians with valuable insights into managing these uncommon cardiac pathologies effectively.

## Case presentation

**Figure 1:**
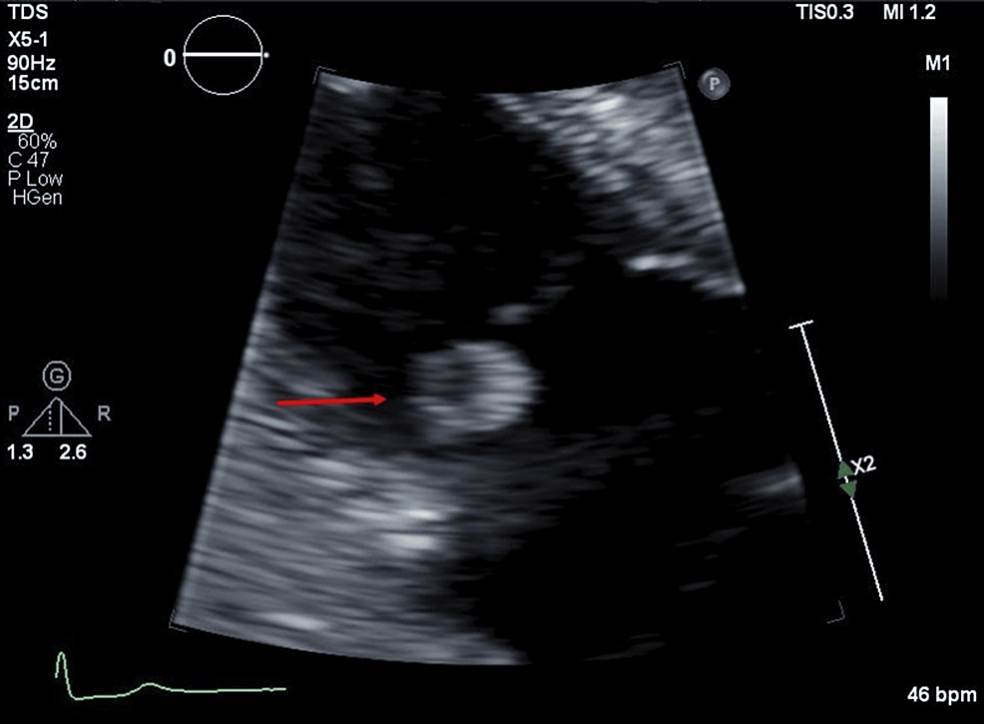
Transthoracic echocardiogram: Right ventricular modified (lateral) apical four-chamber view demonstrates an echogenic mass attached to the anterior tricuspid valve leaflet.

An 81-year-old Caucasian man with a history of coronary artery disease, benign prostatic hyperplasia, and hypertension presented to an outpatient cardiologist with progressive chest pain for the past three to six months. The patient denied any other major symptoms at the time. He was afebrile with a blood pressure of 158/64 mmHg and a heart rate of 76 beats per minute. On physical exam, he had a 2/6 diastolic decrescendo murmur best heard at the right upper sternal border. A TTE showed a degenerative moderate aortic valve regurgitation and a 1.3 cm x 1.2 cm mobile mass seen on the atrial aspect of his tricuspid valve anterior leaflet (Figure 1 and Video 1).

**Video 1:** Transthoracic echocardiogram: Right ventricular modified (lateral) apical view demonstrates a hyperechoic mass attached to the anterior tricuspid valve leaflet.

Given the low clinical suspicion for endocarditis due to the absence of clinical signs typically associated with the condition, such as fever, positive blood cultures, or systemic infection indicators, the mass was further investigated with CMRI, which revealed a round-shaped, oscillating, 1.2 cm x 1.0 cm mass on the atrial side of the anterior tricuspid valve leaflet confirming the presence of a mass detected on TTE (Video 2).

**Video 2:** A balanced steady-state free precession four-chamber view demonstrates a mass attached to the anterior tricuspid valve leaflet (indicated by the red arrow) and signal dephasing consistent with aortic valve regurgitation (indicated by the yellow arrow).

**Figure 2:**
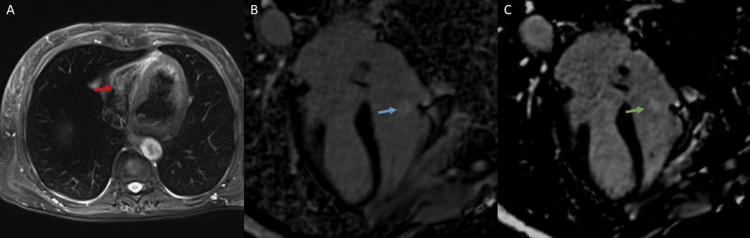
(A) Axial fat-saturated T2-weighted images demonstrate a tricuspid valve mass with hyperintense signal (red arrow). (B) Late gadolinium phase-sensitive inversion recovery sequence displays circumferential enhancement of a tricuspid valve mass (blue arrow). (C) Long inversion time (TI) late gadolinium phase-sensitive inversion recovery sequence demonstrates homogeneous enhancement of a tricuspid valve mass (green arrow).

Furthermore, CMR tissue characterization revealed that the mass was hyperintense on T2-weighted imaging, hyperintense on fat-saturated T2-weighted imaging, and isointense on T1-weighted imaging. On the rest first-pass perfusion images, the mass did not take up significant gadolinium, which likely indicated the lack of significant mass vascularity. In addition, the mass demonstrated no enhancement on early gadolinium-enhanced sequences (EGE), circumferential enhancement on late gadolinium-enhanced sequences (LGE), and homogeneous enhancement on long inversion time (long TI = 550 ms) late gadolinium sequences. The CMR tissue characteristics, size, and location of the mass were most consistent with a PFE (Figure 2).

Based on the progressive nature of the patient’s symptoms with an abnormal stress test, he underwent coronary angiography which revealed severe multi-vessel coronary artery disease. The patient underwent a five-vessel coronary artery bypass graft with aortic valve replacement and resection of the tricuspid valve mass. On pre-operative transesophageal echocardiography (TEE), the anterior tricuspid valve leaflet mass was also visualized on multiple views (Video 3).

**Video 3:** A transesophageal echocardiogram in the low esophageal modified right ventricular view at 132 degrees demonstrates a mobile hyperechoic mass attached to the anterior tricuspid valve leaflet.

**Figure 3:**
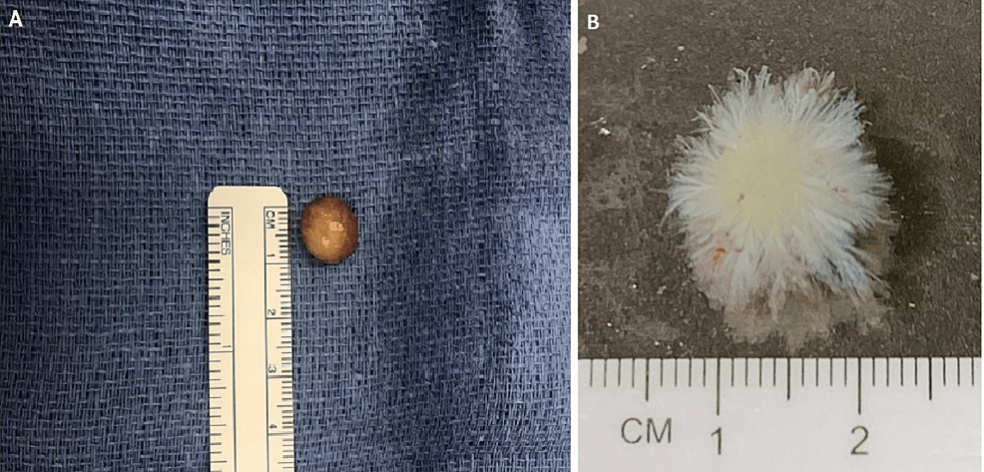
(A) A gross photo of the specimen shows a tan mass measuring 1.6 x 1.5 x 0.8 cm with suggestive papillary/villous structures. (B) A gross photo of the specimen suspended in water to better demonstrate arborizing, thin strands of tan-white tissue arising from a common stalk.

On gross examination, the mass was described as a tan, villous lesion measuring 1.6 x 1.5 x 0.8 cm (Figure 3A). A gross image of the lesion suspended in water displayed arborizing, thin strands of tan-white tissue arising from a common stalk (Figure 3B).

**Figure 4:**
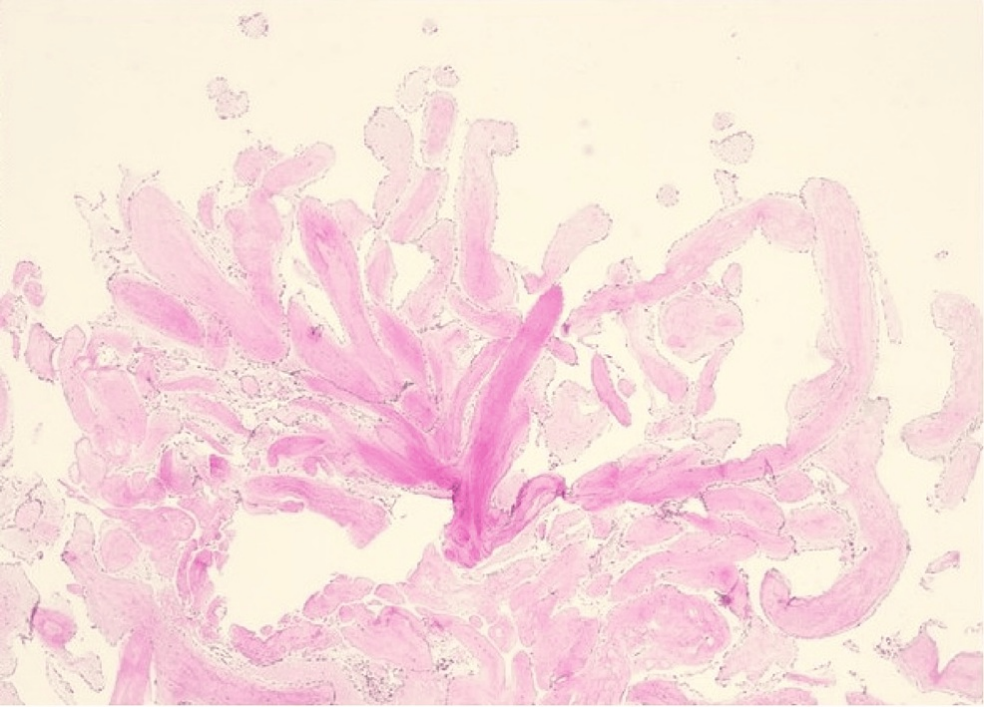
A routine hematoxylin and eosin (H&E) stain at 20x total magnification reveals multiple, branching fronds of paucicellular, avascular fibroelastic tissue lined by a single layer of endocardium. These findings are consistent with a diagnosis of papillary fibroelastoma.

Histologic examination showed multiple, branching fronds of paucicellular, avascular fibroelastic tissue lined by a single layer of the endocardium, findings which were consistent with a diagnosis of PFE (Figure 4).

## Discussion

The incidence of primary cardiac tumors is extremely low, with approximately 1 to 10 cases per 10,000 people per year [2]. PFEs are among the most common benign primary tumors of the cardiac valves, commonly arising from the normal components of the endocardium such as smooth muscle or fibrous tissue. PFEs have a predilection for the left side of the heart, with the aortic valve being the predominant site involved, followed by the mitral valve. Usually, small-sized lesions (<1.5 cm) and PFEs are composed of collagen and elastic tissue fibers lined by endothelium with a short pedicle. These tumors can clinically present with neurologic deficits but are asymptomatic almost 65% of the time [2]. Manifestations of symptomatic right-sided PFEs encompass a spectrum of clinical presentations. These may entail pulmonary embolism, characterized by abrupt onset of dyspnea and chest discomfort; obstructive symptoms, such as peripheral edema and hepatic congestion stemming from tricuspid valve obstruction; and arrhythmias, eliciting palpitations, vertigo, or syncopal episodes. TTE is the most useful tool in the initial evaluation of cardiac masses [3]. The demonstration of a well-demarcated, small, mobile, pedunculated, valvular, or endocardial mass that prolapses into the cardiac chambers during the cardiac cycle is characteristic of a PFE [3]. Furthermore, a characteristic TTE morphologic feature of PFEs, which could help differentiate them from vegetations or thrombi, is the presence of a "speckled/stippled" appearance of peripheral echolucencies in the blood-tumor interface. While TTE remains the quickest and most common imaging modality used for initial cardiac mass evaluation [3], it has several disadvantages, including operator dependence, poor tissue characterization, and, in some cases, acoustic window restrictions in a subset of patients such as obese patients, patients with chest wall deformities, and/or previous cardiac surgery. TEE is frequently subsequently required for a more comprehensive assessment of a cardiac mass, including improved localization of the mass (especially in relation to other cardiac structures) and more accurate mass measurements. Computed tomography (CT) can also provide better characterization but at the expense of exposure to radiation and limited contrast resolution. On the other hand, CMR imaging has excellent contrast resolution and allows for superior tissue characterization. After the administration of gadolinium, PFEs typically display no or little enhancement when images are acquired early after gadolinium administration but increase enhancement if images are obtained after a delay of several minutes [4]. This pattern of delayed enhancement is caused by the content of collagen in PFEs, which is similar in content to infarcted scar tissue. Additionally, cine-MRI allows an assessment of myocardial and valvular function comparable to echocardiography [4].

Our patient underwent TTE as an initial evaluation and subsequently underwent CMR imaging to further differentiate the mass. CMR imaging demonstrated a characteristic PFE enhancement pattern with circumferential enhancement on LGE sequences as well as homogeneous enhancement on long TI LGE sequences. In one study by Giusca et al. [5], the authors reported that malignant cardiac tumors more frequently display an isointense signal in T1 bright blood acquisitions (78% vs. 61%, p = 0.04) and LGE (83% vs. 60%, p = 0.05) compared to benign cardiac tumors. While TTE can provide valuable initial information regarding the location and hemodynamic effects of the mass, CMR is a more valuable tool in establishing the type of cardiac tumor. The advantages of CMR compared to echocardiography are its larger field of view, better spatial resolution, better tissue characterization, lack of attenuation, and ability to image at any prescribed plane [6]. CMRI is, therefore, an appropriate technique for a thorough noninvasive evaluation of cardiac masses and helps distinguish tumors from thrombi [7]. That said, due to its low temporal resolution, CMRI is generally not indicated for the evaluation of valvular vegetations [8]. The unusual location of the tumor initially made us suspect an atrial myxoma on TTE imaging; however, with further CMR imaging, the mass demonstrated features more consistent with a PFE. Subsequent surgical resection and histological examination confirmed that the tumor was a PFE. Tricuspid valve PFE is uncommon, with only very few cases reported in the literature. The American College of Cardiology (ACC) guidelines recommend surgical excision in symptomatic patients who are surgical candidates and an antiplatelet agent in patients who are at risk of surgical removal [9]. Management of asymptomatic patients is still not well-defined.

## Conclusions

PFEs are benign cardiac tumors that are often asymptomatic but can potentially lead to embolic events. Our case highlights the importance of multimodality imaging in the evaluation of cardiac masses. Given the limited data on the management of asymptomatic or incidentally discovered tricuspid valve fibroelastomas, further research is needed to define the optimal management of these patients. That said, for symptomatic patients, patients undergoing cardiac surgery for other cardiac conditions, and those with highly mobile masses, it may be reasonable to consider surgical excision.
